# The safety and efficacy of intra-arterial low-dose tirofiban administration during endovascular therapy in patients with large ischemic core volume

**DOI:** 10.1038/s41598-024-53715-8

**Published:** 2024-02-09

**Authors:** Kwang-Chun Cho, Nak-Hoon Son, So Hyeon Gwon, Jin Wook Choi, Woo Sang Jung

**Affiliations:** 1 Department of Neurosurgery, Yongin Severance Hospital, Yonsei University College of Medicine, Yongin, South Korea; 2 Department of Statistics, Keimyung University, Daegu, South Korea; 3 Department of Radiology, Ajou University Hospital, Ajou University College of Medicine, 164, World Cup-Ro, Yeongtong-Gu, Suwon, 16499 South Korea

**Keywords:** Acute ischemic stroke, Endovascular therapy, Alberta Stroke Program Early CT Score, Tirofiban, Diseases, Neurology

## Abstract

This study aimed to evaluate the safety and efficacy of intra-arterial (IA) administration of low- dose tirofiban during endovascular therapy in patients with large ischemic core volumes on initial brain CT. Patients were divided into two groups based on the use of IA tirofiban. We identified 87 patients (16 and 71 patients in the tirofiban and no-tirofiban groups, respectively) with acute ischemic stroke due to intracranial artery occlusion who underwent endovascular therapy with a low Alberta Stroke Program Early CT scores (2–5). Multivariate logistic regression analysis revealed no association between IA tirofiban administration and serious postprocedural hemorrhagic complications (adjusted odds ratio (aOR), 0.720; 95% confidence interval (CI) 0.099–5.219; p = 0.960), any radiologic hemorrhage (aOR 0.076; 95% CI 0.003–2.323; p = 0.139), or 3-month mortality (aOR, 0.087; 95% CI 0.005–1.501; p = 0.093). However, IA tirofiban was associated with a lower 90-day mRS score (aOR, 0.197; 95% CI 0.015–1.306; p = 0.017) and change of NIHSS compared with baseline (aOR, 0.698; 95% CI 0.531–0.917; p = 0.010). IA tirofiban administration during endovascular therapy in patients with large ischemic core volumes may be effective and safe.

## Introduction

Endovascular therapy (EVT) such as intracranial mechanical thrombectomy (MT) is the first-line treatment strategy for selected patients with acute ischemic stroke (AIS) due to large vessel occlusion (LVO)^1–3^. Moreover, recent studies revealed that even patients with a large ischemic core with an Alberta Stroke Program Early CT Score (ASPECTS) < 6 may benefit from revascularization^4,5^, despite the currently validated eligibility criteria for MT^6^.

Tirofiban is a highly selective glycoprotein IIb/IIIa receptor antagonist, which can effectively block the final stages of the platelet aggregation pathway and prevent subsequent thrombus formation, which can potentially benefit successfully recanalized patients with AIS. Recent studies have reported the safety and efficacy of intra-arterial (IA) tirofiban in LVO stroke patients undergoing MT^7–10^. However, most of the available data consist of patients with a small ischemic core volume, whose ASPECTS ≥ 6 points on the initial CT scan^7–11^. Although IA tirofiban infusion is beneficial in cases involving stent deployment or ongoing thrombus formation, this treatment may increase the risk of bleeding complications^12^, particularly in patients with large ischemic cores.

Thus, we aimed to evaluate the safety and efficacy of the IA administration of low-dose tirofiban during endovascular therapy in patients with a large ischemic core volume on initial CT.

## Methods

### Patients

We retrospectively collected data from patients with AIS who underwent EVT at a single tertiary hospital between 2017 and 2022. The inclusion criteria for patients in this study were as follows: (1) patients who presented to the emergency department with AIS due to internal carotid artery (ICA), middle cerebral artery (MCA; M1 and M2), or tandem large vessel occlusion and underwent EVT; and (2) patients with an ASPECTS between 2 and 5 on the initial brain CT performed in the emergency department and analysed using the automated software tool (RAPID ASPECTS software (Vesion 4.9; iSchemaView, Menlo Park, Calif). RAPID ASPECTS software performs a series of operations to generate an automated ASPECTS evaluation^13^.

The exclusion criteria were as follows: (1) poor quality of the initial brain CT resulting in low reliability of the ASPECTS analysed using the RAPID program; (2) unknown mRS score at the three-month follow-up after EVT; and (3) stroke caused by arterial dissection. The study was approved by the Ajou University Hospital Institutional Review Board (AJOUIRB-DB-2023-097), and the requirement for written informed consent was waived because of the retrospective study design. All methods were performed in accordance with the relevant guidelines and regulations^3,6^.

### Endovascular therapy and IA tirofiban use

Three neurointerventionalists performed EVT. All had over 10 years of experience in neurointervention and could perform EVT proficiently. All procedures were performed under local anaesthesia. Intravenous (IV) heparin was mandatory to maintain the activated clotting time between 200 and 300 s during the procedure, except in subjects treated with IV tissue plasminogen activator (t-PA). The EVT procedure was chosen at the discretion of the neurointerventionalists. Stent retrieval and contact aspiration were routinely used. In cases of failed EVT for LVO, rescue treatments, including emergency stent placement, balloon angioplasty, or tirofiban infusion, were administered. Tirofiban was administered only to patients with failed EVT based on the following criteria: (1) residual stenosis ≥ 70% in the occlusion site after thrombectomy with forward blood flow not maintained by modified Treatment in Cerebral Infarction (mTICI) ≥ 2b, (2) rescue treatment with stenting or balloon angioplasty, and (3) reocclusion after the first reperfusion. Treatment involved 0.5 mg (2 mL) tirofiban diluted in 8 mL of normal saline, which was injected at an infusion rate of 1 mL/min. On follow-up angiography immediately and 10 min after IA infusion, the same protocol was used if additional tirofiban was required. The total IA tirofiban infusion ranged from 0.5 to 2.0 mg.

### Data collection and outcomes

We analysed the clinical characteristics, including age, sex, NIHSS score on admission, and baseline mRS scores. The baseline ASPECTS was determined using the RAPID program based on the initial non-contrast brain CT performed in the emergency department^14^. The scores of all patients were then reviewed, and if a score was unreliable, it was determined by consensus of the two neurointerventionalists. Follow-up brain CT was performed to evaluate hemorrhagic complications immediately and 12–24 h after EVT. Aetiology of LVO was determined by angiographic diagnosis, as previously reported^15^.

Clinical efficacy outcomes included the mRS score at 90 days and the changes in NIHSS compared with baseline at 7 days (or discharge if earlier). Technical efficacy outcomes included successful reperfusion and postprocedural early reocclusion within 48 h of follow-up CTA or MRA. Based on the final angiography, successful reperfusion was defined as mTICI grade 2b (partial filling ≥ 50% territory) or 3 (complete reperfusion). Safety outcomes included the incidence of symptomatic intracranial hemorrhage within 48 h, any radiologic intracranial hemorrhage, and mortality within 90 days of treatment. Symptomatic intracranial hemorrhage was defined as parenchymal haematoma type 1 and 2 and/or thick subarachnoid hemorrhage (SAH) with or without intraventricular hemorrhage (modified Fisher grade 3 or 4 of SAH) as per the Second European-Australasian Acute Stroke Study (ECASS II) criteria^16^.

### Statistical analyses

Descriptive statistics were used for the between-group comparisons of patient characteristics and outcomes. Categoric variables were analysed using *x*^2^ tests or Fisher exact tests and were presented as percentages. Continuous variables were analysed using Student *t* test or Mann–Whitney U test and presented as median and interquartile range. Multivariate logistic regression analysis was performed to evaluate the efficacy and safety of tirofiban. Variables with p < 0.15 in the bivariate analysis were included in the multivariate logistic regression analysis. All statistical analyses were performed using the R statistical and computing software (R Foundation for Statistical Computing; Vienna, Austria), and statistical significance was set at p < 0.05.

### Ethics approval

Ethical approval was obtained from the relevant Institutional Review Board (AJOUIRB-DB-2023-097). The requirement for written informed consent was waived because of the retrospective nature of this study.

## Results

### Baseline characteristics of patients

**Figure 1:**
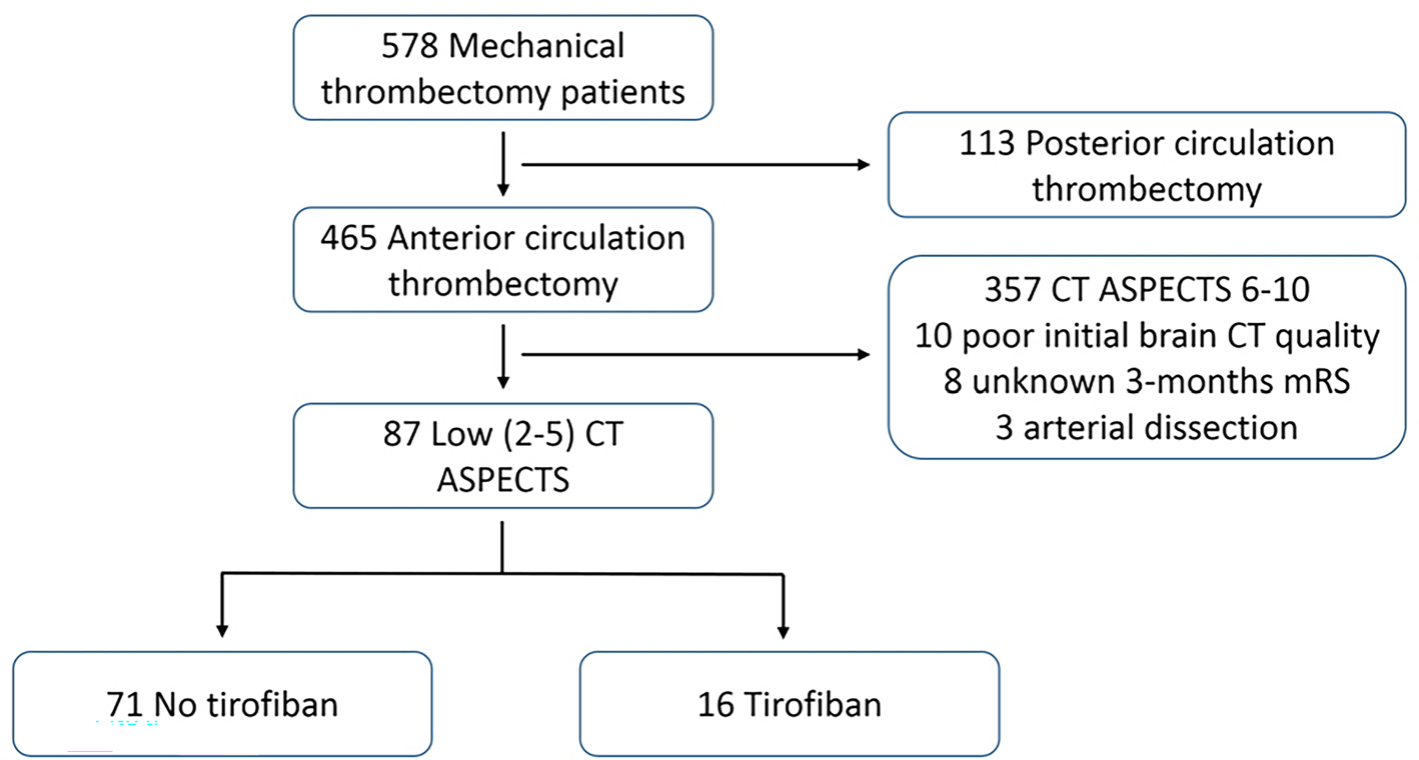
Study flow chart. *ASPECTS* Alberta Stroke Program Early CT Score, *mRS* modified Rankin Scale.

We analysed 87 patients who underwent EVT with low initial ASPECTS on brain CT. Sixteen patients were treated with tirofiban (tirofiban group), whereas 71 patients were not (no-tirofiban group) (Fig. 1). Table 1 presents the baseline patient characteristics. Compared with the no-tirofiban group, the tirofiban group had significantly more cases of intracranial atherosclerotic stenosis-related occlusion (ICAS-O) (9.9% vs. 81.2%, p < 0.001), higher rates of rescue treatment (1.4% vs. 31.2%, p < 0.001), and longer procedure times (45 min vs. 75 min, p = 0.035). There was no significant difference in the baseline NIHSS scores between the two groups.

**Table 1:** Basline characteristics of patients between two groups.

	No tirofiban (n = 71)	Tirofiban (n = 16)	P value
Sex (male)	41 (57.7)	13 (81.3)	0.081
Age (median) (IQR)	74 (62–82)	63.5 (55.5–70.5)	0.048
Smoking	13(18.3)	7 (43.7)	0.045
Hypertension	38 (53.5)	12 (75.0)	0.116
DM	11 (15.5)	3 (18.7)	0.716
Hyperlipidemia	15 (21.2)	7 (43.7)	0.107
A-fib	38 (53.5)	2 (12.5)	0.002
CAD	11 (15.5)	1 (6.2)	0.452
Previous stroke	15 (21.2)	5 (31.2)	0.511
IV tPA	20 (28.1)	3 (18.7)	0.543
NIHSS on admission (median) (IQR)	19 (17–22)	16 (13.5–21.5)	0.053
Baseline mRS (median) (IQR)	0 (0–1)	0 (0–1)	0.717
Target artery occlusion site			0.013
Distal ICA	27 (38.0)	1 (6.2)	
MCA	37 (52.1)	10 (62.5)	
Tandem occlusion	7 (9.9)	5 (31.2)	
Underlying ICAS	7 (9.9)	13 (81.2)	< 0.001
Rescue therapy	1 (1.4)	5 (31.2)	< 0.001
Onset to door time (min, median) (IQR)	210 (90–465)	187.5 (120.5–432.5)	0.698
Procedure time (min, median) (IQR)	45 (25–90)	75 (52.5–95)	0.035
First line recanalization device			0.217
Stent	43 (60.6)	12 (75.0)	
Suction catheter	26 (36.6)	3 (18.7)	
Combined	2 (2.8)	1 (6.3)	

*DM* diabetes mellitus, *A-fib* atrial fibrillation, *CAD* coronary artery disease, *tPA* tissue plasminogen activator, *NIHSS* National Institutes of Health Stroke Scale, *ICAS* intracranial atherosclerotic stenosis, *IQR* inter quartile range.

### Safety and efficacy outcomes

Table 2 presents the efficacy and safety outcomes of tirofiban treatment. There was no significant difference in symptomatic hemorrhagic complications (26.7% vs. 25.0%; unadjusted OR 0.621 (0.121–3.197, 95% CI); p = 0.956), any radiologic hemorrhage (49.3% vs. 31.2%; unadjusted OR 0.486 (0.051–4.763, 95% CI); p = 0.536), or 90 day mortality (23.9% vs. 12.5%; unadjusted OR 0.388 (0.054–3.307, 95% CI); p = 0.367) between the two groups. There was also no significant difference in the successful reperfusion rate (80.3% vs. 75.0%; unadjusted OR 0.995 (0.179–5.519, 95% CI); p = 0.995) or early reocclusion rate within 48 h on follow-up CTA or MRA (4.2% vs 18.7%; unadjusted OR 5.213 (0.949–28.827, 95% CI); p = 0.057). Multivariate logistic regression analysis revealed no association between IA tirofiban and serious hemorrhage (adjusted OR [aOR], 0.720; 95% confidence interval [CI] 0.099–5.219; p = 0.960), radiologic hemorrhage (aOR, 0076; 95% CI 0.003–2.323; p = 0.139), or mortality within three months (aOR: 0.087; 95% CI 0.005–1.501; p = 0.093).

**Table 2:** Efficacy and safety outcomes between two groups.

	No tirofiban (n = 71)	Tirofiban (n = 16)	Unadjusted OR (95% CI)	Adjusted^a^ OR (95% CI)	P value Unadjusted/adjusted
Clinical efficacy outcome					
mRS score at 90d (median) (IQR)	5 (4–5)	4 (3–4.5)	0.651 (0.031–3.522)	0.197 (0.015–1.306)	0.025/0.017
Change in NIHSS compared with baseline (median) (IQR)	0 (–4 to 2)	–3 (–6.5–0.5)	0.875 (0.770–0.995)	0.698 (0.531–0.917)	0.019/0.010
Technical efficacy outcome					
Successful reperfusion (mTICI2b-3)	57 (80.3)	12 (75)	0.995 (0.179–5.519)	1.066 (0.088–12.924)	0.995/0.960
Early reocclusion within 48 h	3 (4.2)	3 (18.7)	5.213 (0.949–28.827)	1.172 (0.055–25.206)	0.057/0.919
Safety outcome					
Symptomatic hemorrhage within 48 h	19 (26.7)	4 (25.0)	0.621 (0.121–3.197)	0.720 (0.099–5.219)	0.956/0.960
Any radiologic hemorrhage	35 (49.3)	5 (31.2)	0.486 (0.051–4.763)	0.076 (0.003–2.323)	0.536/0.139
90 days mortality (mRS 6)	17 (23.9)	2 (12.5)	0.388 (0.054–3.037)	0.087 (0.005–1.501)	0.367/0.093

*mRS* modified Rankin Scale, *NIHSS* National Institutes of Health Stroke Scale, *IQR* inter quartile range, *mTICI* modified Treatment In Cerebral Infarction.

^a^Variables used as adjustment factors : age, smoking, A-fib, target artery occlusion site, underlying ICAS, rescue therapy, procedure time.

In the clinical efficacy outcome aspects, the median (IQR) 90-day mRS score was significantly lower in the tirofiban group than in the no tirofiban group (4(3–4.5) vs 5(4–5); p = 0.025), and the median (IQR) change in NIHSS compared with baseline also differed significantly between the tirofiban and no tirofiban group (− 3 (− 6.5 to − 0.5) vs 0 (− 4 to 2); p = 0.019, respectively). After adjustment, IA tirofiban was associated with a lower 90-day mRS score (aOR, 0.197; 95% CI 0.015–1.306; p = 0.017) and change in NIHSS compared with baseline (aOR, 0.698; 95% CI 0.531–0.917; p = 0.010).

## Discussion

Recent studies have demonstrated that EVT could improve functional outcomes in patients with a large ischemic core volume without affecting the incidence of symptomatic hemorrhage^4,5,17^. Similarly, previous studies reported that the use of low-dose IA tirofiban during EVT did not increase the risk of ICH, symptomatic ICH, or 3-month mortality^18–20^. Our results indicate that low-dose IA tirofiban administration did not increase the risk of symptomatic hemorrhagic complications, radiologic intracranial hemorrhage, or 3-month mortality in AIS patients undergoing EVT, even in patients with a large ischemic core volume on the initial CT scan. In addition, IA tirofiban was associated with lowering the 90-day mRS score and improving the baseline NIHSS score. However, this did not significantly reduce the incidence of early reocclusion.

Tirofiban is a glycoprotein IIb/IIIa inhibitor that suppresses platelet aggregation in a dose-dependent manner. It has a short half-life, with platelet function normalising after 4 h^21,22^. Given these pharmacokinetics, tirofiban can be used safely during certain types of EVT, such as angioplasty or stenting for stenotic lesions, from the perspective of hemorrhagic complications. However, the use of tirofiban as rescue therapy during EVT produced varied results. One possible explanation could be the difference in the administration methods of the drug across studies. Kellert et al. suggested that IV tirofiban increases the risk of fatal intracranial hemorrhage and poor functional outcomes in patients treated with EVT^23^. In contrast to IV tirofiban, other studies have shown that low-dose IA tirofiban administered during EVT is safe^7–10^. Jang et al. reported that low-dose IA tirofiban did not increase the risk of bleeding in patients who underwent EVT with IV tPA^7^. On the contrary, Yang et al. demonstrated that IV tirofiban was not associated with any increase of sICH and IA tirofiban increased rate of sICH for patients with small core volume^24^. Another possible explanation for conflicting results could be associated with ischemic core volume rather than the administration methods.

In this study, we found that low-dose IA tirofiban did not increase the risk of bleeding in patients with an initial large ischemic core volume who underwent EVT. These results are consistent with those of previous studies on tirofiban, which did not consider the patients’ initial ischemic core volume^7–11^. The safety of tirofiban may be attributed to the several advantages of administering it via IA. IA tirofiban can be administered at a lower dose than intravenously. In the study by Kellert et al., tirofiban was administered IV, infused at 0.4 mg/kg/min for 30 min, followed by a continuous infusion of 0.1 mg/kg/min for 48 h. The total tirofiban dose was 18 mg for a 60-kg adult. Our dose (0.5–2.0 mg) was smaller than the IV tirofiban dose, and the drug could be administered in a target artery. Consequently, we speculated that the dose was an important factor, and that low-dose tirofiban was feasible during EVT in patients with a large ischemic core volume. However, it is also possible that the initial non-contrast brain CT scan did not accurately reflect the ischemic core volume and overestimated it.

In this study, the rate of successful reperfusion (mTICI = 2b/3) was lower, whereas the rate of early reocclusion within 48 h of follow-up imaging after EVT was higher in patients treated with low-dose IA tirofiban, though without statistical significance. This may be explained by the fact that the use of tirofiban was at the discretion of the treating interventionalists, who were prone to use tirofiban in patients with a high possibility of reocclusion after achieving partial recanalization of the occluded arteries during EVT, meaning that not all arteries were recanalized or ultimately achieved good reperfusion. Thus, the higher rate of early reocclusion and lower rate of successful reperfusion could have occurred due to the much higher rate of patients with underlying atherosclerotic stenosis of the occluded arteries in the tirofiban group. This bias likely explains the seemingly paradoxical study results, and further RCTs are needed to investigate whether tirofiban can improve reperfusion status.

Interestingly, the functional outcomes were better in patients treated with tirofiban, despite slightly lower rate of successful reperfusion. Previous studies have hypothesised that tirofiban can improve patient prognosis^11,15,19,25,26^. Even if the major arterial occlusion recanalizes after EVT, microvascular thrombosis may continue to occur at distal sites. Given their topographical localisation to microvessels distributed throughout the ischemic territory, the in-situ formation of microthrombi and microembolisms during thrombectomy procedures may contribute to the reduction of post-ischemic flow and infarct progression^27^. Studies have shown that glycoprotein IIb/IIIa antagonists are effective in preventing and treating microthrombosis and accelerating recovery from AIS^27–29^. Therefore, we speculate that tirofiban may improve functional outcomes by improving the reperfusion status of the microvasculature and maintain the reflow status.

Once the safety of treatment modalities is established, clinicians prioritize functional outcomes over radiological outcomes. Therefore, based on the results of this study, we anticipate that low-dose IA tirofiban could be a viable option for patients with low ASPECTS.

This study had several limitations. Firstly, the retrospective design of this study exposes it to gaps in prospective data collection, such as collecting mRS scores at three months for all patients. Secondly, the sample size was limited. In particular, only 16 patients received IA tirofiban. Thirdly, the use of IA tirofiban was at the discretion of the neurointerventionalist, and there might have been a selection bias. Specifically, neurointerventionalists may have decided to use IA tirofiban only if they consider it safe. Finally, the dose and use of tirofiban in this study were inconsistent, which necessitates further investigation to determine the optimal tirofiban treatment protocol for patients with AIS who are treated with EVT.

## Conclusions

Our results indicate that low-dose IA tirofiban administration during EVT is safe and effective in patients with large ischemic core volume (low ASPECTS) and could be used to improve functional outcomes. To the best of our knowledge, this is the first study to assess the safety and efficacy of tirofiban in patients with AIS with a large ischemic core volume.

## Data Availability

The datasets generated during and/or analysed during the current study are available from the corresponding author on reasonable request.
